# The Importance of Thinking Multivariately When Setting Subscale Cutoff Scores

**DOI:** 10.1177/00131644211023569

**Published:** 2021-07-14

**Authors:** Edward Kroc, Oscar L. Olvera Astivia

**Affiliations:** 1 University of British Columbia, Vancouver, British Columbia, Canada; 2 University of Washington, Seattle, Washington, USA

**Keywords:** measurement, cutoff scores, multivariate analysis, reliability, classification

## Abstract

Setting cutoff scores is one of the most common practices when using scales to aid in classification purposes. This process is usually done univariately where each optimal cutoff value is decided sequentially, subscale by subscale. While it is widely known that this process necessarily reduces the probability of “passing” such a test, what is not properly recognized is that such a test loses power to meaningfully discriminate between target groups with each new subscale that is introduced. We quantify and describe this property via an analytical exposition highlighting the counterintuitive geometry implied by marginal threshold-setting in multiple dimensions. Recommendations are presented that encourage applied researchers to think jointly, rather than marginally, when setting cutoff scores to ensure an informative test.

## Introduction

The use of measures and scales to classify individuals is one of the oldest and perhaps most controversial uses of testing to this day (Kaplan & Saccuzzo, 2017; Loewenthal & Lewis, 2018; Thorndike et al., 1991). From the early days of the Standord-Binet test for mental capacity to modern computerized adaptive testing, relying on tests to classify people into groups or rank them according to some trait or (latent) ability has been of major interest. To understand the scope of this prevalence, consider the following. According to the College Board (2019), in the United States alone 2.2 million students took the SAT and more than 8 million students took a test from the SAT suite of tests during the 2018-2019 school year. For the GRE, more than a half million people took the test in the past year alone (Educational Testing Services, 2019). Large-scale educational testing has become a critical component of a high school student’s aim to be selected to a prestigious college, university, or other postsecondary program, and the decision hangs, at least in part, on whether the student is above or below a certain cutoff score on those tests, usually derived from their quantile position with respect to other students (Soares, 2015).

Given the relevance that testing has in helping classify, rank, or diagnose individuals, various approaches have been developed to find optimal decision points along a scale above (or below) which an individual must score before she or he is categorized (Cizek, 2006; Habibzadeh et al., 2016; Kaftandjieva, 2010). The immediate question then becomes how should this threshold be determined? Since many of the conclusions derived from a test administration depend on whether or not the threshold has been chosen as correctly as possible to match the intended uses of the test, this question is among the most salient.

In the majority of cases, the process of setting thresholds depends on whether a test is norm-referenced or criterion-referenced (Hambleton & Novick, 1973). Norm-referenced tests compare a test-taker’s responses with the responses of their peers in order to create a distribution of scores along which each respondent can be located. Criterion-reference tests specify a cutoff score in advance, which does not change irrespective of the performance of the test-takers.

Perhaps one of the most widely used classification systems of methods used to set cutoff scores is the Jaeger (1989) system, which divides them into test-centered or examinee-centered. Test-centered methods seek to establish threshold values based on the characteristics of the test, the items or the scoring process, whereas examinee-centred rely on the particular characteristics of the test-takers to set the cutoff scores. In spite of the importance of considering the characteristics of test-takers during the scoring process, test-centered methods are more widely used and we will offer a brief summary of some methods that are popular among researchers and test-developers (Kane, 1998; Lewis & Lord-Bessen, 2017). For a more extensive discussion of examinee-centered methods please consult Kaftandjieva (2010).

Judgment-based methods encompass procedures such as those described in Angoff (1971), Ebel (1972), Jaeger (1982), and Nedelsky (1954). The commonality among these (and other) methods is that the impressions of subject-matter experts and experienced researchers in the domain area play a role in deciding which items or test scores should be used as thresholds. They usually intersect with other types of statistical analyses, but the emphasis is placed on the subjective evaluation and agreement among expert judges regarding what the cutoff score should be.

There are also a variety of statistically oriented techniques where the emphasis is on the score distribution and cutoffs are set based on whether or not this distribution has certain properties. One of the oldest and perhaps most popular methods still being used within the applied psychometric literature is selecting the threshold value to correspond to a certain number of standard deviation (*SD*) units above or below the mean, usually under the assumption of normally distributed data. Traditionally, a value beyond 2 *SD* units is selected to emphasize the fact that individuals are categorized on the basis of their extreme scores, which differentiates them from what a “typical” score respondent would look like. Scales such as the Ages and Stages Questionnaire (Bricker et al., 1988), the Minnesota Multiphasic Personality Inventory (Schiele et al., 1943) and the Early Development Instrument (Janus & Offord, 2007) have relied on this method, at least in their initial conceptualization. A closely related method that is widely used in the health sciences is setting cutoff values based on percentiles (Loewenthal & Lewis, 2018). In this scenario, the threshold values would also come from a normative sample, but the difference from the *SD* units described above is that a particular cumulative probability is used as a decision point, as opposed to a value away from the mean. Both methods are intrinsically related, though, since one could switch from one approach to the other under the assumption that the random distribution’s parameters are known. For instance, if X is a random variable defining the responses to a particular scale and $X\sim N(\mu ,\sigma^{2})$ , whether one chooses a threshold value 2 *SDs* above the mean or the 97.5th percentile, the cutoff point would be (nearly) the same.

As computational power became more readily available, more sophisticated approaches also became popular among researchers interested in developing new measures. A particularly popular one, which comes from signal detection theory, is the receiver operating characteristic curve (ROC). In essence, ROC curve analysis attempts to solve a binary classification problem by finding the optimal points that balance the classifier’s true positive rate (also known as probability of detection or sensitivity) and the false negative rate (also known as probability of false alarm or specificity; Fawcett, 2006; Zou et al., 2016). Ideally, the point that maximizes the area under the curve also offers the best balance between sensitivity and specificity, thus offering researchers with an optimal cutoff value to use as a threshold. Since the popularization of ROC curve analysis in psychometrics came after the use of the percentile or the *SD* method, many scales have collected further validity evidence by analyzing new data using this technique, such as the Beck Depression Inventory-II (Beck et al., 1996) or the revised version of the Ages and Stages Questionnaire (Squires et al., 1997). For a more exahustive overview of how to use ROC curve analysis, please refer to Fawcett (2006).

Item response theory is perhaps the most advanced theoretical framework devoted to the development and analysis of tests and measures. Item response theory approaches attempt to relate the probability of item responses to hypothesized, true latent traits (usually designated by the Greek letter θ ), with the assumption being that items whose parameters best provide information about the latent θ would be the most optimal ones in the development and scoring of a scale. Based on these item parameters, one can also locate scores within the test that maximize the information it contains through the test information function. Lord and Novick (1968) offer a procedure to set up threshold values either at the item level or the test level to minimize the loss of information and maximize discrimination among test-takers.

Irrespective of which method is employed to set the threshold values, their practical and applied use is very similar across scales. Once a respondent scores above or below the predefined cutoff, she or he is assigned to a particular category to complement the process of assessment or to aid in some diagnostic procedure. As we will unpack in the following sections, such a process imposes a particular geometry on the resulting categories. When these categories are the product of cutoff scores from many subscales set *independently*, we will show that such assessments quickly lose their value; that is, lose their ability to meaningfully discriminate members of one category from another. Even when a categorization is based on as few as 4 subscale scores, as many as 25% of sample subjects will be unreliably classified (see section Empirical Demonstrations). Such tests thus may be considered to have questionable discriminatory reliability simply because of how they create their diagnostic categorizations *marginally* over multiple dimensions/subscales of the target phenomenon.

## Theoretical Framework

### Reliably Classified Individuals

Throughout this article, the method of setting threshold values based on being above or below the mean plus or minus a certain number of *SD* units will be employed as our primary working example, as we consider it to be the easiest one to understand (and one of the oldest ones still in use). Nevertheless, it is important to point out that similar conclusions would be found if different methods, as discussed in the Introduction, were implemented.

Anytime one employs an imperfect measurement (i.e., test) to classify individuals into groups, some sample individuals will eventually be misclassified. Intuitively, the less reliable a test, the less reliable the classifications. We mean to invoke both the intutitive and the technical meaning of *reliablity* here, as defined classically in, for example, Lord and Novick (1968). Indeed, low reliablity of a test necessarily implies that individuals with the same (latent) true score will likely be assigned substantially different observed scores by the test. The less reliable the test, the more variation we will observe in these observed scores for otherwise interchangeable sample respondents (interchangeable in that they all share a true score/ability). And by the same token, a perfectly reliable test would perfectly distinguish sample individuals according to their (latent) true scores/abilities; thus, classification arising from such a test would be error-free.

We do not intend to dwell here on the exhausting number of ways to *quantify* reliablity that have been proposed and argued in the literature, as our point only requires the acknowledgement that a test that is not perfectly reliable in the population will inevitably lead to misclassification when people are split into groups that are supposed to reflect some *latent ability/trait* according to their *observed scores* on the test. Then, those individuals whose observed scores fall near the *boundary* of these group definitions are the most likely to be misclassified. A few examples will help us illustrate this.

Let Y be a test with a single (sub)scale, composed of an arbitrary number of items and, for ease of exposition, assume that Y is well approximated as Y~N(0,1) . Also, let X be a test with two subscales $\left(X_{1},X_{2}\right)$ , each composed of an arbitrary number of items, and again for ease of exposition, assume that $(X_{1},X_{2})\sim N\left(\begin{array}{cc} \left[\begin{array}{c} 0\\ 0 \end{array}\right], & \left[\begin{array}{cc} 1 & \rho \\ \rho & 1 \end{array}\right] \end{array}\right)$ . Note that ρ denotes the correlation coefficient between both subscales. For these hypothetical tests, we will consider a sample respondent to “pass the test” if they score above −1 on all subscales; otherwise, they “fail the test.” Thus, our testing classification scheme creates two disjoint groups according to a sample unit’s observed scores.

**Figure 1.**
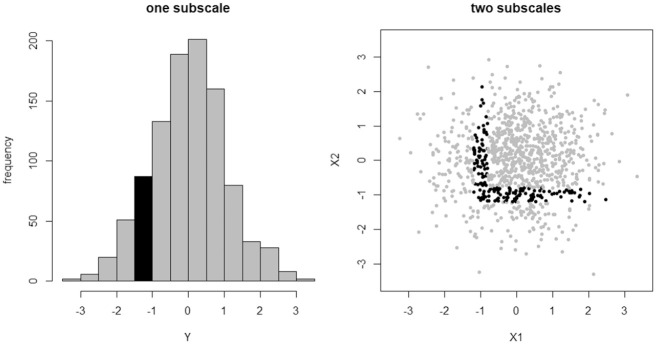
Unreliably classified individuals (black points) from a one subscale or two subscale (uncorrelated) test where cutoffs have been set independently. Approximately 9% of sample individuals are unreliably classified under test Y , whereas approximately 16% are unreliably classified under test X .

Under test Y , we theoretically expect about 84% of sample individuals to pass the test; that is, to achieve an observed score greater than −1. If test reliablity is less than perfect though, some of these sample individuals will be misclassified. In particular, those who achieve an observed score close to the threshhold of −1 are the most likely to be misclassified. Depending on how reliable we consider this test to be, individuals with observed scores falling inside the interval [−1−δ,−1+δ] for some δ>0 can be considered *unreliably classified*. For our purposes, the exact value of δ , and how it is computed, are immaterial. One could arrive at many reasonable values for it depending on how one chooses to quantify reliablity of the test. The point is that (at least one) such a functional δ>0 exists, as this allows us to identify those individuals for whom we feel the least confident in our classifications. For this illustration, we will set δ=0.2 . The first graphic in Figure 1 then shows the approximately 9% of sample individuals who are unreliably classified under test Y from a simulated sample of 1,000 respondents.

Now consider the analogous situation under test $X=(X_{1},X_{2})$ when ρ=0 ; that is, a two subscale test where those subscales are uncorrelated. Under this test, we theoretically expect about 70% of sample individuals to pass the test; that is, to achieve an observed score greater than −1 on each subscale simultaneously. If the test is less than perfectly reliable though, we will again be most suspicious of those individuals whose scores place them near the boundary defined by these cutoffs. For δ=0.2 again, this region of unreliably classified individuals is illustrated in the second graphic in Figure 1 and it contains approximately 16% of all sampled individuals. Notice that this region looks like the corner of a box, a geometrical observation that will be very important for us in the following sections.

What we see here is that a test with two subscales and independently set cutoff scores creates a considerably *higher* proportion of unreliably classified individuals than an analogous test on only one (sub)scale. The situation gets worse as we continue to add subscales (see the next section), until eventually virtually the entire sample will be unreliably classified.

**Figure 2.**
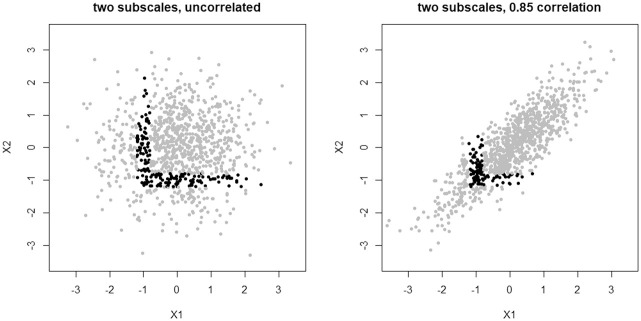
Two tests, each composed of two subscales with ρ=0 or ρ=0.85 . Cutoffs have been set independently. Unreliably classified individuals (black points) make up approximately 16% of the sample under the first test, whereas only about 10% of the sample is unreliably classified under the second test.

There are two ways one could hope to address this problem. One would change how the subscales relate to each other; the other would change how the pass/fail boundary is defined. Considering the first possibility, we will see in the next section that inducing correlation between subscales can slow but not ultimately prevent the problem. Indeed, this can be visualized as in Figure 2. Here, the previous two subscale test X is compared with another two subscale test where those subscales are now highly correlated, ρ=0.85 . For this second test, only 10% of sample individuals are now reliably unclassified (using the same choice of δ=0.2 as before), close to but still larger than the proportion of sample individuals unreliably classified by the single (sub)scale test Y . This result makes intuitive sense since as ρ→1 , the two subscales collapse into a single scale. Nevertheless, aside from this pathological scenario, the proportion of unreliably classified individuals will continue to grow without bound as the number of subscales increases (see the next section).

The second option then is where we can hope to avoid this problem. In the next section, we state and prove a theorem that quantifies just how informative a test can be as the number of subscales to be (independently) thresholded increases, assuming normal data. More precisely, we show that as the number of subscales increases, a test determined by independently setting thresholds at each subscale necessarily loses all of its ability to disciriminate between “passing” and “failing” individuals; that is, all sample respondents will become unreliably classified, indistinguishable up to ordinary measurement error. This suggests that the way forward requires setting cutoff scores *jointly* across subscales, rather than marginally/independently.

### Theoretical Results

We first present our main result and its proof. In the subsequent subsections, we unpack the practical meaning of this result for applied practice, with a focus on the geometrical implications of threshold-setting in higher dimensions (i.e., with many subscales). We focus on the case of multivariate normality for two important reasons. First, because it appears that (either explicitly or implicitly) this is the standard assumption made regarding the joint distribution of subscale scores in most testing situations. And second, because the requisite mathematics are reasonably accessible under a multivariate normal structure.

**Theorem 1.** *Let $X_{d}=(X_{1},\ldots ,X_{d})$ be a d -dimensional normal random variable with mean vector $\mu_{d}$ and covariance matrix $\Sigma_{d}$ . Denote*

$$q_{c}:=Pr\left(D_{M}(X_{d}-\mu_{d})\leq c\right),$$

*where c>0 and $D_{M}(X_{d}-\mu_{d})$ is the d -dimensional Mahalanobis distance from $X_{d}$ to $\mu_{d}$ *:

$$D_{M}(X_{d}-\mu_{d})=\sqrt{{\left(X_{d}-\mu_{d}\right)}^{T}\Sigma_{d}^{-1}(X_{d}-\mu_{d})}.$$

*Also denote*

$$p_{c}:=Pr\left(\overset{d}{\underset{i=1}{⋂}}\{|X_{i}-\mu_{d}|\leq c_{i}\}\right),$$

*where $c:=(c_{1},\ldots ,c_{d})$ is a fixed vector with all components positive. Then if $c_{d}↛0$ and $c_{d}↛\infty$ as d→∞ , and $\underset{d>0}{\sup}\left(\underset{i\neq j}{\sup}|C\mathrm{orr}(X_{i},X_{j})|\right)<1$ , then $q_{c}/p_{c}\to 0$ as* d→∞ .

In one dimension, $q_{c}$ is simply the probability that a normal X lies within cσ of its mean. In an arbitrary number of dimensions d , this is the probability that $X_{d}$ lies within an ellipsoid with principal axes and orientation determined by the elements of $\Sigma_{d}$ . When $\Sigma_{d}=I_{d}$ , this ellipsoid is the d -dimensional unit sphere. One can view $\Sigma_{d}$ (or, more precisely, $\Sigma_{d}^{1/2}$ ) as tranforming the sphere into an ellipsoid via directional scalings along the principal axes defined by the correlations between the components of $X_{d}$ . In this way, Mahalanobis distance is the ordinary Euclidean distance scaled by the square root of the inverse covariance matrix $\Sigma_{d}^{-1/2}$ .

At the same time, in one dimension, $p_{c}$ is simply the probability that a normal X lies within $c_{1}$ of its mean. In an arbitrary number of dimensions d , this is the probability that $X_{d}$ lies within a d -dimensional box centred at $\mu_{d}$ with sidelengths given by $2c_{i}$ . It is critical to recognize that in one dimension, $q_{c}$ and $p_{c}$ denote the *same probabilities*; that is, setting $c_{1}=c\sigma$ , we have $q_{c}=p_{c}$ . However, these quantities capture probabilities over very different regions (shapes) in higher dimensions (recall the corner of the box in two dimensions of the previous section). As we will see, boxes and ellipsoids capture very different pieces of space in a high number of dimensions, and their difference encapsulates the essence of unreliable classification. We exploit this geometric reality to prove our theorem: that the ratio $q_{c}/p_{c}\to 0$ as the number of dimensions/subscales d→∞ , which should be viewed as an analytical rephrasing of our claim that the proportion of unreliably classified individuals approaches 100% as the number of subscales increases. We will unpack this in sections Geometry of Cutoff Scores and Empirical Demonstrations to see how this analytical result implies that multidimensional tests that set independent cutoff scores on each of their subscales to classify individuals lose all their value to meaningfully discriminate between individuals as the number of subscales increases.

*Proof of Theorem 1.* We first convert $q_{c}$ into a more useful expression. Define $Z_{d}=\Sigma_{d}^{-1/2}(X_{d}-\mu_{d})$ . Then we have:

$$\begin{array}{c} D_{M}(X_{d}-\mu_{d})=\sqrt{Z_{d}^{T}Z_{d}}\\ =\sqrt{\sum_{i=1}^{d}Z_{i}^{2}} \end{array}$$

where $Z_{i}\sim N(0,1)$ . That is, the square of the Mahalanobis distance follows a chi-square distribution with degrees of freedom equal to the dimension of the vector $X_{d}$ . So:

$$\begin{array}{c} q_{c}=\mathrm{Pr}(D_{M}(X_{d}-\mu_{d})\leq c)\\ \;=\mathrm{Pr}(D_{M}^{2}(X_{d}-\mu_{d})\leq c^{2})\\ \;=\mathrm{Pr}\left(\sum_{i=1}^{d}Z_{i}^{2}\leq c^{2}\right). \end{array}$$

Note that, applying the central limit theorem and then standardizing, one recovers the classical result that $q_{c}\to 0$ as d→∞ .

**Figure 3.**
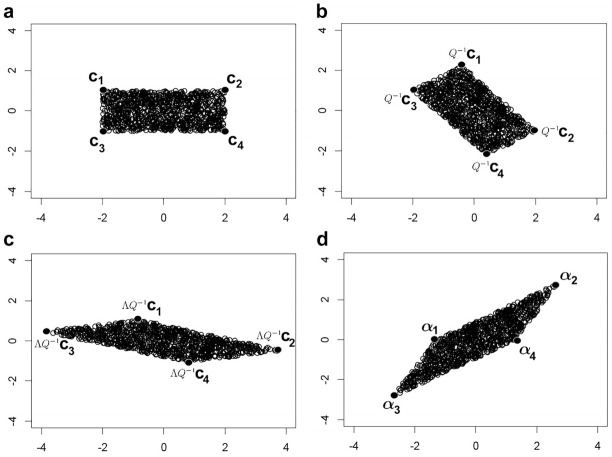
Transforming (a) a box/rectangle into a parallelepiped/parallelogram, via the spectral decomposition of $\Sigma_{d}^{-1/2}=Q\Lambda Q^{-1}$ , (b) rotation, (c) scaling, (d) undo rotation. For this example, one transforms the box defined by $\left\{-2\leq x_{1}\leq 2\}\cap \{-1\leq x_{2}\leq 1\right\}$ via the covariance matrix $\Sigma_{d}=\left[\begin{array}{cc} 1 & 0.5\\ 0.5 & 2 \end{array}\right]$ .

It is in fact also true that $p_{c}\to 0$ , a fact that will increase our workload as we want to show that the ratio $q_{c}/p_{c}\to 0$ . To see how, we again standardize so that $Z_{d}=\Sigma_{d}^{-1/2}(X_{d}-\mu_{d})$ , where $Z_{d}\sim \mathrm{MVN}(0,I_{d})$ . Now, the event of interest

(1) $$\overset{d}{\underset{i=1}{⋂}}\{|X_{i}-\mu_{d}|\leq c_{i}\}$$

describes a box in d dimensions determined by the collection of vertices defined by the boundaries of the inequalities. There are precisely $2^{d}$ such vertices, for example, $\left(c_{1},c_{2},\ldots ,c_{d}\right)$ , $\left(-c_{1},c_{2},\ldots ,c_{d}\right)$ , $\left(c_{1},-c_{2},\ldots ,c_{d}\right)$ , $\left(-c_{1},-c_{2},\ldots ,c_{d}\right)$ , and so on. Enumerate these vertices as $c_{1},c_{2},\ldots ,c_{2^{d}},$ and define $\alpha_{k}:=\Sigma_{d}^{-1/2}(c_{k}-\mu_{d})$ . These new vertices define a parallelepiped, and since the transformation of $c_{k}-\mu_{d}$ into $\alpha_{k}$ is linear, invertible, and continuous, the event (1) on $X_{d}$ is equivalent to the event of falling on or inside this parallelepiped on $Z_{d}$ (see Figure 3). To take advantage of the marginal independence structure of $Z_{d}$ however, we need to simplify this geometry.

Define

(1) $$\alpha :=\left(\underset{1\leq i\leq d}{\max}|\alpha_{i1}|,\underset{1\leq i\leq d}{\max}|\alpha_{i2}|,\ldots ,\underset{1\leq i\leq d}{\max}|\alpha_{\mathrm{id}}|\right).$$

Consider the event

(1) $$\overset{d}{\underset{i=1}{⋂}}\{|Z_{i}|\leq \alpha_{i}\},$$

where $\alpha =(\alpha_{1},\ldots ,\alpha_{d})$ , $Z_{i}\sim N(0,1)$ . Just as in (1), this event describes a box in d dimensions determined by the collection of vertices defined by the boundaries of the inequalities. Moreover, this box contains the parallelepiped obtained by transforming (1), by definition of α . Thus,

(1) $$\mathrm{Pr}\left(\overset{d}{\underset{i=1}{⋂}}\{|X_{i}-\mu_{d}|\leq c_{i}\}\right)\leq \mathrm{Pr}\left(\overset{d}{\underset{i=1}{⋂}}\{|Z_{i}|\leq \alpha_{i}\}\right),$$

and we are now in a position to take advantage of the simple structure of $Z_{d}\sim \mathrm{MVN}(0,I_{d})$ . To whit,

(1) $$\begin{array}{c} p_{c}=\mathrm{Pr}\left(\overset{d}{\underset{i=1}{⋂}}\{|X_{i}-\mu_{d}|\leq c_{i}\}\right)\\ \;\leq \mathrm{Pr}\left(\overset{d}{\underset{i=1}{⋂}}\{|Z_{i}|\leq \alpha_{i}\}\right)\\ \;=\overset{d}{\underset{i=1}{\Pi }}(\Phi (\alpha_{i})-\Phi (-\alpha_{i}))\\ \;=\overset{d}{\underset{i=1}{\Pi }}(2\Phi (\alpha_{i})-1), \end{array},$$

where Φ(·) is the cumulative distribution function of the univariate standard normal. Since $\frac{1}{2}<\Phi (\alpha_{i})<1$ for all finite positive $\alpha_{i}$ , this product is strictly decreasing in d . Moreover, since $c_{d}↛\infty$ , this product converges to zero. More precisely, if a positive sequence $\left\{a_{1},a_{2},\ldots \right\}$ of numbers in (0,1) does not converge to 1, then $\log(a_{i})$ does not converge to 0 by continuity. Thus, $\underset{n\to \infty }{\lim}\sum_{i=1}^{n}\log(a_{i})=-\infty$ , and (by continuity of the exponential) $\overset{\infty }{\underset{i=1}{\Pi }}a_{i}=0.$

Now, to prove that the ratio $q_{c}/p_{c}\to 0$ , we will slightly adapt the previous argument to obtain a lower bound for $p_{c}$ . Just as we were able to construct a box in the $Z_{d}$ coordinates that contained the event that $X_{d}$ lies within another box, we may instead construct a box in the $Z_{d}$ coordinates that itself is contained in the event that $X_{d}$ lies within the original box. Recall that $\alpha_{k}$ denoted the $2^{d}$ vertices that defined the parallelepiped in $Z_{d}$ . Rather than containing this object in a larger (regular) box, we instead consider the largest (regular) box that lies entirely *inside* the parallelepiped (see Figure 3). Denote the $2^{d}$ vertices of this box by $\beta_{k}$ .

We need to ensure that this box does not degenerate as d→∞ ; that is, we would be in trouble if this box collapsed into a lower than full dimensional object. This can only happen, however, if some of the eccentricities of the parallelepiped approach zero as d→∞ . This is not possible though as long as $c_{d}↛0$ , $c_{d}↛\infty$ , and $\underset{d>0}{\sup}\left(\underset{i\neq j}{\sup}|\mathrm{Corr}(X_{i},X_{j})|\right)<1$ , as we have assumed in the statement of Theorem 1. This first condition ensures that the original box does not degenerate (i.e., collapse into a lower than full dimensional object), so by continuity of our transformations, the parallelepiped in $Z_{d}$ also cannot degenerate. The second condition on the sequence $\left\{c_{d}\right\}$ ensures that the parallelepiped in $Z_{d}$ does not become pointy and narrow without bound, so the relative lengths of the principal axes of the parallelepiped (i.e., the eccentricities) remain bounded away from infinity. Notice that we used this second fact to prove that $p_{c}\to 0$ , but we did *not* care in that argument if our boxes in $X_{d}$ or in $Z_{d}$ degenerated in the first sense. The condition that $\underset{d>0}{\sup}\left(\underset{i\neq j}{\sup}|C\mathrm{orr}(X_{i},X_{j})|\right)<1$ ensures that our subscales can never become perfectly correlated, which would result in another less than full dimensional object.

Now, we may construct a lower bound on $p_{c}$ as before:

(1) $$\begin{array}{c} p_{c}=\mathrm{Pr}\left(\overset{d}{\underset{i=1}{⋂}}\{|X_{i}-\mu_{d}|\leq c_{i}|\}\right)\\ \;\geq \mathrm{Pr}\left(\overset{d}{\underset{i=1}{⋂}}\{|Z_{i}|\leq \beta_{i}\}\right)\\ \;=\overset{d}{\underset{i=1}{\Pi }}(2\Phi (\beta_{i})-1). \end{array}$$

Note that since $\beta_{i}$ can never equal 0 (because the box defined by the $\beta_{k}$ vertices is nondegenerate), this lower bound is nonzero for all d . Thus,

(1) $$\frac{q_{c}}{p_{c}}\leq \frac{q_{c}}{\overset{d}{\underset{i=1}{\Pi }}(2\Phi (\beta_{i})-1)}$$

As we have already seen, the numerator can be expressed as

(1) $$q_{c}=\mathrm{Pr}\left(\sum_{i=1}^{d}Z_{i}^{2}\leq c^{2}\right),$$

where $Z_{i}\sim N(0,1)$ are i.i.d. This is the probability that a random d -vector $Z_{d}\sim \mathrm{MVN}(0,I_{d})$ is contained in the d -ball of radius c . Since the maximum of the density function of the standard multivariate normal in any number of dimensions d is always less than 1, this probability is bounded by the ordinary (Lebesgue) volume of the d -ball of radius c (see section Geometry of Cutoff Scores). Thus,

(2) $$\begin{array}{c} \frac{q_{c}}{p_{c}}\leq \frac{\frac{\pi^{\frac{d}{2}}}{\Gamma \left(\frac{d}{2}+1\right)}c^{d}}{\overset{d}{\underset{i=1}{\Pi }}(2\Phi (\beta_{i})-1)}\\ \;\underset{\sim }{<}\frac{2e\pi^{\frac{d-1}{2}}c^{d}d^{-\frac{d+1}{2}}}{\overset{d}{\underset{i=1}{\Pi }}(2\Phi (\beta_{i})-1)}, \end{array}$$

where the “ $\underset{\sim }{<}$ “ notation signifies that the inequality may require a positive constant that does not depend on any quantities of interest (notably, d ); this standard analytical notation (e.g., see Tao, 2011) eliminates the need to consider lower order asymptotic remainders. See section Geometry of Cutoff Scores for more details on these analytics.

For the denominator of (2), we apply the mean value theorem to find

(2) $$2\Phi (\beta_{i})-1=\frac{1}{\sqrt{2\pi }}\int_{-\beta_{i}}^{\beta_{i}}e^{-\frac{x^{2}}{2}}\mathrm{dx}=\frac{2\beta_{i}}{\sqrt{2\pi }}\cdot e^{-\frac{b_{i}^{2}}{2}},$$

for some $b_{i}\in (-\beta_{i},\beta_{i})$ . Since the $\beta_{i}$ s are uniformly bounded away from zero and from infinity, we find that

(2) $$2\Phi (\beta_{i})-1\geq \frac{m}{2}\cdot e^{-\frac{s^{2}}{2}},$$

for each i , where $s:=\underset{d>0}{\sup}\left(\underset{1\leq i\leq d}{\sup}\beta_{i}\right)<\infty$ and $m:=\underset{d>0}{\inf}\left(\underset{1\leq i\leq d}{\inf}\beta_{i}\right)>0$ . Consequently, we bound (2) as follows:

(2) $$\begin{array}{l} \frac{q_{c}}{p_{c}}≲\frac{2e\pi^{\frac{d-1}{2}}c^{d}d^{-\frac{d+1}{2}}}{\prod_{i=1}^{d}\left(2\Phi (\beta_{i})-1\right)}\\ \;\;\;\;\;\;\leq \frac{2e\pi^{\frac{d-1}{2}}{\left(2c\right)}^{d}d^{-\frac{d+1}{2}}}{m^{d}\exp\left(-\frac{ds^{2}}{2}\right)}\\ \;\;\;\;\;\;\;≲r^{d}\cdot d^{-\frac{d+1}{2}}, \end{array}$$

for some positive constant r . Regardless of the value of r , this quantity goes to zero.

### Geometry of Cutoff Scores

The consequences of Theorem 1 for the discriminatory power of a test defined by setting cutoff scores in multiple subscales demands a closer consideration of the ambient geometry. While practitioners are generally quite comfortable with intuiting univariate phenomena, this intuition can severely breakdown in higher dimensions.

We first reiterate that the quantities $q_{c}$ and $p_{c}$ are equivalent in a single dimension. In higher dimensions however, $q_{c}$ captures the multivariate normal probability of occupying an ellipsoid, while $p_{c}$ captures the multivariate normal probability of occupying a box, the shape that is necessarilly produced when one sets cutoff scores independently in each subscale. To help develop a geometric intuition for these higher dimensional structures, we consider in detail the simplest cases when the ellipse/ellipsoid is simply a circle/sphere, and the box is simply a square/cube.

In d -dimensional Euclidean space, the traditional Euclidean (closed) d -ball centered at the origin of radius r is defined as

(3) $$B_{d}(0,r):=\{x\in R^{d}:|x|=\sqrt{x_{1}^{2}+\cdots +x_{d}^{2}}\leq r\}.$$

In two dimensions, this is all points on or in the circle of radius r centred at the origin, and in three dimensions, this is all points on or in the sphere of radius r centred at the origin. Another important geometric object is the regular (closed) d -box centred at the origin of sidelength 2c , defined as

(4) $$C_{d}(0,2c):=\{x\in R^{d}:|x_{1}|\leq c,\ldots ,|x_{d}|\leq c\}.$$

In two dimensions, this is all points on or in the square of sidelength 2c centred at the origin, and in three dimensions, this is all points on or in the cube of sidelength 2c centred at the origin.

**Figure 4.**
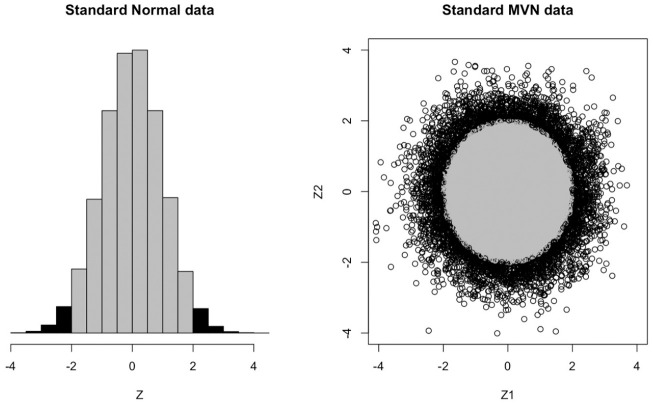
(a) Approximately 95% of the probability density lies within two units of zero of the univariate standard normal. (b) Approximately 86% of the probability density lies within two units of zero of the bivariate standard normal.

Notice that $B_{1}(0,r)=C_{1}(0,2r)$ ; that is, the one-dimensional ball and cube are the same object, simply an interval. This is the biggest hint that our univariate intuition will not be sufficient for multidimensional phenomena. Indeed, while it is perfectly intuitive that most of the density of a univariate normal lies near its mean/mode, as we increase the number of dimensions, more and more of the limited probability density (which must always integrate to 1) must disperse over “larger” sets in Euclidean space. Figure 4 illustrates the situation for one and two dimensions. Here, we illustrate that while in one dimension, approximately 95% of the probability density falls within 2 *SD*s of the mean/mode, in two dimensions, this probability falls to approximately 86%. Moreover, the amount of density that necessarily falls near the boundary created by these cutoffs necessarily increases, reflected in the fact that the ratio $q_{c}/p_{c}$ decreases. In multiple dimensions then, we must better understand the interplay between balls and boxes.

**Figure 5.**
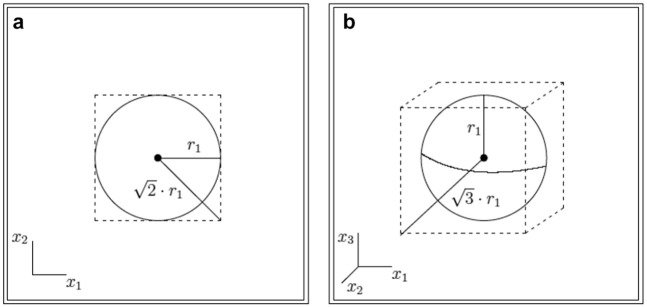
(a) Circle (2-ball) of radius $r_{1}$ contained inside a square (regular 2-box) of sidelength $2r_{1}$ . (b) Sphere (3-ball) of radius $r_{1}$ contained inside a cube (regular 3-box) of sidelength $2r_{1}$ . In general, by the Pythagorean theorem, there is no regular d -box of smaller diagonal than $\sqrt{d}\cdot r_{1}$ that contains the d -ball of radius $r_{1}$ .

It is a classical fact of analytic geometry that any d -ball of radius r can be contained inside a d -box of sidelength 2r . The situation is depicted in Figure 5 for two and three dimensions. In general, this fact is a direct consequence of the Pythagorean theorem. That is, for any $x\in B_{d}(0,r)$ , the d -ball centred at the origin (without loss of generality) of radius r , we know by (3) that

(4) $$\sqrt{x_{1}^{2}+\cdots +x_{d}^{2}}\leq r.$$

Squaring both sides of this inequality, we see that $x_{i}^{2}\leq r^{2}$ must hold for every 1≤i≤d , else this sum of d nonnegative numbers would exceed $r^{2}$ . But this means that x must also be contained in the box of sidelength 2r centred at the origin by (4); i.e., $x\in C_{d}(0,2r)$ since

(4) $$|x_{1}|\leq r,\ldots ,|x_{d}|\leq r$$

for all $x\in B_{d}(0,r)$ .

For completeness, we show the classical but rather counterintuitive result in high dimensional geometry that, as the number of dimensions grows, the volume of the unit d -ball approaches zero while the volume of the smallest regular d -box containing the unit d -ball approaches infinity. Indeed, the volume of the unit d -ball is given by the equation

(4) $$V\mathrm{ol}(B_{d}(0,R))=\frac{\pi^{\frac{d}{2}}}{\Gamma \left(\frac{d}{2}+1\right)}R^{d},$$

where Γ(·) is the regularized gamma function defined as:

(4) $$\Gamma \left(\frac{d}{2}+1\right)=\int_{0}^{\infty }t^{\frac{d}{2}}e^{-t}\mathrm{dt}.$$

By Stirling’s formula, this function is asymptotically equivalent to

(4) $$\Gamma \left(\frac{d}{2}+1\right)\sim \sqrt{\pi d}{\left(\frac{d}{2e}\right)}^{\frac{d}{2}}.$$

Thus,

(4) $$\begin{array}{c} V\mathrm{ol}(B_{d}(0,1))=\frac{\pi^{\frac{d}{2}}}{\Gamma \left(\frac{d}{2}+1\right)}\\ \sim \frac{2e\pi^{\frac{d-1}{2}}}{d^{\frac{d+1}{2}}}\to 0, \end{array}$$

since $d^{d}$ grows far more rapidly than $r^{d}$ for any fixed positive constant r . On the other hand, the smallest regular d -box containing $B_{d}(0,1)$ is the hypercube centred at the origin of sidelength 2: $C_{d}(0,2)$ . But clearly,

(4) $$V\mathrm{ol}(C_{d}(0,2))=2^{d}\to \infty .$$

It is easy to see that these results can be extended to balls and regular boxes of nonunit scale. Specifically, $V\mathrm{ol}(B_{d}(0,R))\to 0$ for any fixed R , and $V\mathrm{ol}(C_{d}(0,2R))\to \infty$ when $R>\frac{1}{2}$ , $V\mathrm{ol}(C_{d}(0,1))\to 1$ , and $V\mathrm{ol}(C_{d}(0,2R))\to 0$ when $R<\frac{1}{2}$ . It is important to notice though that even when the volume of the hypercube goes to zero with increasing dimension, it does so at a *much slower* rate than the volume of the ball, by virtue of the gamma function in the denominator of $V\mathrm{ol}(B_{d}(0,R))$ .

One major consequence of these facts is that most of the volume of the regular d -box *must be contained near its corners*. A glance back at Figure 5 should be helpful. As the number of dimensions grows, the unit d -ball encompasses less and less of the volume of its smallest enclosing d -box. But since the d -ball still touches each face of the d -box at its center, the only way the two volumes can diverge so massively is if the d -box is essentially “all corners” as the dimension grows.

These results immediately generalize to ellipsoids and irregular boxes, so long as the lengths of their principal axes and, respectively, sidelengths remain uniformly bounded (this was our condition that $c_{d}↛\infty$ in Theorem 1). That is, the volume of such an ellipsoid must go to zero as the dimension grows, and most of the volume of the smallest irregular d -box containing this ellipsoid is concentrated near its corners. This generalization is a direct consequence of the fact that ellipsoids are simply images of balls under invertible linear transformations.

**Figure 6.**
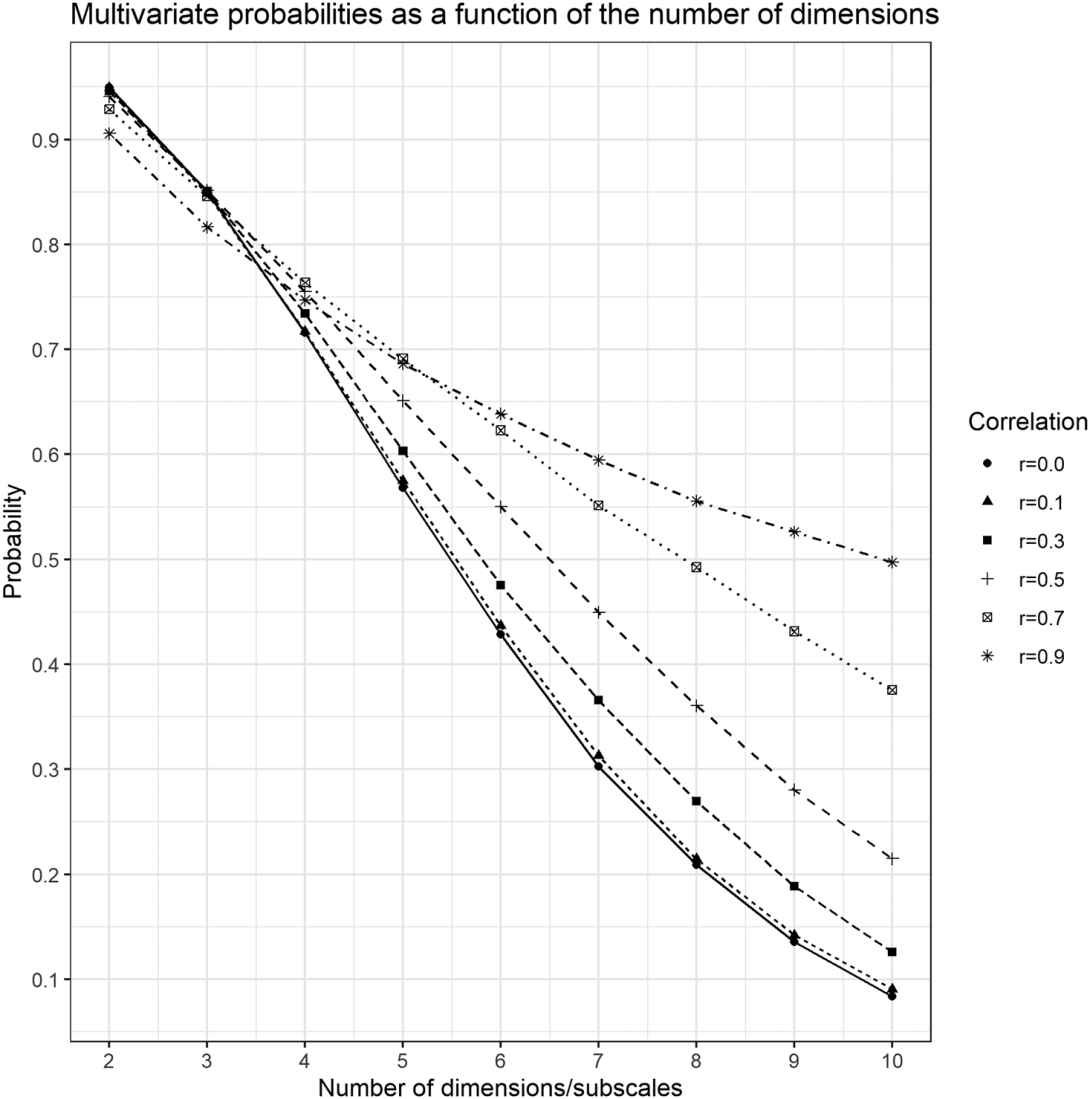
Percentage of reliably classified individuals as a function of the number of subscales. That is, ratios of the multivariate normal probability of falling inside the ellipsoid over the multivariate normal probability of falling inside the smallest box containing the ellipsoid as a function of the number of dimensions. The ellipsoids and boxes are determined by hypothetical threshold ±2 z scores from the mean vector. Different dotted lines and shapes correspond to multivariate normal distributions with different degrees of correlation ( ρ=0.0,0.1,0.3,0.5,0.7 , and 0.9 ). All dimensions/subscales are assumed to be equicorrelated.

What is important for us though, and what Theorem 1 establishes, is that a similar geometry holds when we measure the volume of an ellipsoid or box using a multivariate normal measure, rather than the standard notion of Euclidean volume (i.e., Lebesgue measure). Indeed, Theorem 1 shows that the multivariate normal probability of falling inside a d -ellipsoid or a d -box centered at the mean approaches zero as d increases, and that the ratio $q_{c}/p_{c}\to 0$ . It is this final piece that ensures that *more and more of the multivariate normal mass of the box lies in its corners (i.e., outside the largest d -ellipsoid contained inside the box) as the dimension increases* (see Figures 4, 5, and 6). Three important implications for multivariate threshold-setting follow:

1. In direct contrast with the univariate setting, more and more of the sample data from a multivariate normal distribution will fall *away* from the “typical respondent” (i.e., the d -ball centred at the mean/mode vector of the distribution) as the dimension increases.
2. Consequently, if sample individuals are required to satisfy many marginal thresholds *simultaneously* and *independently* in order to be classified as belonging to some normative group of interest (i.e., in order to “pass the test”), then most of these individuals will appear near the boundary of at least one of these thresholds.
3. In the presence of naturally occuring measurement error (or, equivalently, under a less than perfectly reliable test), these individuals inside the normative group and near the boundary are *indistinguishable* from individuals outside the normative group. Thus, such a test loses all its discriminatory power of classification.

Implication 3. is the most relevant for our practical recommendations. If we imagine a collection of subscales designed to assess aptitude of some particular latent trait θ , then (3) implies that *most individuals who are classified as belonging to the group of interest are virtually indistinguishable from those who just missed classification*. Put another way, the more subscales with thresholds one must cross, the less discriminating the test actually is. That is, most classified individuals are essentially exchangeable with nonclassified individuals, and this problem gets worse with the inclusion of more thresholded subscales. This suggests several problems of fairness and efficacy of setting thresholds marginally for many subscales simultaneously.

It is important to note that while we have concentrated on normative sets defined by regular, symmetric thresholds, the same results hold for irregular, asymmetric thresholds (e.g., $c_{1}\leq X_{1}\leq c_{2}$ with $\left|c_{1}|\neq |c_{2}\right|$ ). The same proof that we have provided holds in this case with only minor modifications. Furthermore, Implications 1, 2, and 3 will also hold if only a single threshold is set for each subscale; for example, $X_{1}\geq c_{1}$ and $X_{2}\geq c_{2}$ .

### Empirical Demonstrations

Theorem 1 demonstrates that any normative set constructed via independent thresholding of subscales will become less discriminating as the number of subscales increases. This is a reflection of the ambient geometry of higher dimensional space, where most of the mass of a normative set is forced to lie near the “corners” of the set, as defined by the subscale thresholds. Consequently, as the number of subscales increases, most individuals become very atypical in the sense that they must occupy a point in space that is far away from the mean/mode for multivariate normal phenomena. Moreover, if the normative set is defined as those individuals who fall within a certain distance from the marginal means of the subscales, then the size of this set will shrink without bound as the number of subscales increases. Take, for instance, a scenario where a battery of screening psychological tests are implemented on a group of participants with the aim of grouping them in “at risk” versus “not at risk” categories. One would expect that only a certain percentage of participants would exhibit enough markers or symptoms to be classified as belonging to the clinical group, the “true” population percentage of people exhibiting the pathology or characteristic of interest. If decision thresholds classifying participants as “at risk” versus “not at risk” did not consider how the various tests interact (i.e., setting them scale by scale as opposed to jointly), one would expect to have more participants classified as “at risk” as the number of tests or subscales increases arbitrarily, missing the true population percentage of participants who exhibit the pathology or characteristic of interest. Counterintuitively, having more tests or more subscales classifying participants does not necessarily imply these participants are being more reliably classified, unless the process of setting thresholds was calibrated with the aim to make one decision based on the joint relationship between tests, as opposed to one decision based on simply their individual, marginal structures.

To showcase the practical implications of Theorem 1 and the geometrical explanations that follow, Figure 6 presents the decreasing probabilities of $q_{c}/p_{c}$ from six different types of multivariate normal distributions. To locate oneself within the context of the examples of the previous section, the *x*-axis corresponds to the number of subscales in a hypothetical test and the *y*-axis is the probability of falling within two *SD* units of the mean (i.e., the thresholds would be at the *z* scores at ±2 ). The focal point of the simulation is to contrast the naive understanding of reliably classifying individuals by setting thresholds marginally, subscale by subscale, with the actual mathematical structure of this action implied in higher dimensions. Univariately, setting thresholds at ±2 implies that 95% of respondents would be classified as belonging to the normative group and only the 5% of those in the extreme tails of the distribution would not. However, when multiple dimensions (i.e., subscales) with respective thresholds are at play, that 95% group of respondents shrinks so that fewer and fewer people end up being classified in the normative group.

Figure 6 illustrates the proportion of people in the normative group that are clearly distinguishable (i.e., reliably classified) from those outside the normative group—the multivariate normal volume of the ball divided by the multivariate normal volume of the smallest box containing the ball, $q_{c}/p_{c}$ —as a function of the number of subscales. This is the proportion of individuals who fall near the mean/mode of the distribution, away from the “corners” created by the multiple thresholds. In the context of some latent trait θ , Figure 6 shows how discriminating the test of the latent trait is as a function of the number of marginally thresholded subscales. Regardless of the correlation between subscales, this discrimination is quite high for 2 or 3 subscales, but even at 4 subscales, about 25% of the normative group is essentially exchangeable with individuals outside the normative group in terms of their value of θ . That is, about 25% of the normative sample falls in the “corners” of the 4-box created by the marginal thresholds and so are about as atypical of respondents as individuals who fall outside this box. Contrast this with the univariate situation depicted in Figure 5(a) where only a very small fraction of normative individuals are indistinguishable from those outside the group (i.e., those individuals with z scores very close to the gray/black cutoff).

As the number of thresholded subscales increases, Figure 6 clearly illustrates that fewer and fewer people are reliably classified by the test as more respondents will be forced into the “corners” of the “pass” and “fail” groups. These respondents will not be reliably classified when the test is subject to measurement error. Moreover, the lack of discriminatory power for marginally thresholded tests only worsens as test reliability is compromised.

The degree of correlation between subscales also plays an important role in the ability to reliably classify participants, with higher correlations slowing the rate of unreliable classification as the number of subscales grows. This mathematical fact makes good practical sense too, since one can easily argue that a test composed of two highly correlated subscales has about as much ability to reliably classify individuals as a single subscale test. Regardless, Theorem 1 guarantees that no amount of correlation will be enough to overcome the problem eventually, given enough subscales. Figure 6 shows that by the time one reaches 10 subscales, a test with highly correlated subscales ( ρ=0.9 ) will still unreliably classify about half of the normative group. For the case of independent subscales, virtually the entire normative group will be indistinguishable from individuals who fall outside of it (i.e., the upper bound on the probability of reliably classifying participants is about 0.1).

## Conclusions and Recommendations

Given the frequent use of tests and measures as tools that aid in the classification of respondents, we believe it is important to highlight the differences implied by the traditional method of setting thresholds marginally (i.e., each subscale at a time) versus setting them jointly. From the theoretical results presented and the test scenarios explored, we believe a few important lessons should be highlighted and brought into consideration for applied researchers who may be interested in either developing their own scales or interpreting existing ones. We have summarized these lessons in Implications 1, 2, and 3 of section Geometry of Cutoff Scores and reiterate the lessons here.

First, if a scale has no subscales or a joint decision is not needed to aid in classifying or diagnosing, then setting thresholds univariately bears little influence in the final decision and the results presented here do not necessarily apply. The shape and properties of the marginal distributions would be the only relevant ones in this case. If, however, more than one subscale is used in the decision process, then it is important to remind test users and developers that the probability of selection is always less than or equal to the one implied by each subscale independently. Theorem 1 highlights this issue by pointing out the fact that, for example, even for the well–known case where 95% of the probability of a normal distribution is contained within 2 *SDs*, that probability goes to 0 as the number of dimensions grows. Implications 1 and 2 of section Geometry of Cutoff Scores summarize this problem as the fact that more sample respondents will necessarily fall far away from the “typical” respondent as the dimension increases. Therefore, we would like to encourage researchers to consider the decision process *multivariately* as opposed to in separate univariate pieces. Marginal thinking is how the majority of cutoff values are currently set, but this necessarily ignores joint dependency between the subscales and creates the possibility of entirely untenable diagnostics.

Moreover, the correlations among the subscales affects the probability of classification. In general, larger correlations among the simulated subscales implies a slower rate of decrease in the probability of reliable classification. Therefore, when deciding on a cutoff value (irrespective of the method in which this cutoff value is selected), it is important to keep in mind the correlations among the subscales and to adjust the thresholds accordingly. A potential approach that could be explored would be to use multivariate generalizations to univariate approaches that consider more than just the marginal structure of the subscales. One could, for instance, rely on the centroid of the distribution and the variance-covariance matrix so that thresholds could be set in terms of Mahalanobis distances and then see which combination of coordinates (i.e., sample individuals) correspond to the Mahlanobis distance of choice. Methods that incorporate both marginal and joint information simultaneously are welcome to tackle this issue.^1^

Applied practitioners should expect an inherent amount of measurement error to be present in any testing situation. Too much measurement error often results in unreliable testing procedures, thus motivating the push for the creation of formally *reliable* scales. What our Theorem 1 demonstrates however, is that in the context of independent subscale thresholding, such tests will necessarily lose their discriminatory power to meaningfully categorize individuals as the number of subscales increases, regardless of how formally *reliable* the underlying measurements/subscales are. One natural way to address this intrinsically maladaptive feature is to switch to a more thoughtful process of threshold setting, one that does not always set thresholds on different subscales independently.

## Notes

1 We would like to thank an anonymous reviewer for providing this insight.
